# Th1/Th2 Differentiation and B Cell Function by the Atypical PKCs and Their Regulators

**DOI:** 10.3389/fimmu.2012.00241

**Published:** 2012-08-06

**Authors:** Pilar Martin, Jorge Moscat

**Affiliations:** ^1^Department of Vascular Biology and Inflammation, Fundación Centro Nacional de Investigaciones Cardiovasculares Carlos IIIMadrid, Spain; ^2^Sanford-Burnham Medical Research InstituteLa Jolla, CA, USA

**Keywords:** atypical PKCs, B cells, T cells, Th1, Th2, adaptive immune responses, asthma

## Abstract

The members of the atypical Protein Kinase Cs (aPKC) kinase subfamily, PKCζ and PKCλ/ɩ, as well as their adapters, p62 and Par-6, form part of the PB1-domain-containing group of signaling regulators. Both adapters serve to locate through heterotypic interactions the aPKCs into the NF-κB and cell polarity pathways, respectively. Both signaling cascades have been critically implicated in T cell function *in vitro* and *in vivo*. The analysis of gene-knockout (KO) mice deficient in the different PB1 molecules is providing more definitive information on the actual role that the aPKCs and other PB1-containing molecules play in B cell biology and T cell polarity, survival, and differentiation toward the different effector lineages *in vivo* and at the cellular *ex vivo* level. Here we discuss recent data generated from the analysis of KO mice linking the control of cell polarity by PKCλ/ɩ and PKCζ, their adapter p62, and the Par-4 inhibitor, in the control of B and T cell signaling and differentiation. Altogether, these genetic and biochemical evidences reveal the existence of a PB1-orchestrated signaling network that acts to control Th2 differentiation *in vitro* and *in vivo*, and the gene transcriptional programs that are essential during the B cell maturation and function and Th2 differentiation.

## Introduction

It is well known that cells from the adaptive immune system need proper activation of the nuclear factor κB (NF-κB) for their function and development. NF-κB provides necessary mediators for the survival of immature T and B cells in antigen receptor-mediated negative and positive selection of non-reactive clones in thymus and bone marrow, respectively. NF-κB activation is also necessary for mature lymphocyte differentiation and function (Hayden et al., 2006; Schulze-Luehrmann and Ghosh, 2006). However, uncontrolled activation of NF-κB can induce cancer development, autoimmune, and chronic inflammatory diseases as a result of exacerbated lymphocyte function (Karin and Greten, 2005). The Protein Kinase Cs (PKCs) family is an important piece in the puzzle of NF-κB-mediated cell activation and several studies on recent years have demonstrated that proteins of this family are potent mediators of antigen receptor downstream signaling in B and T cells. Hence, these proteins are key in both, the development and the control of innate and adaptive immune responses. Since there are three different families of PKCs; classical PKCs (PKCα, βI, βII, γ), novel PKCs (PKCδ, ε, η, þθ), and atypical PKCs (aPKCs; PKCζ, λ/ɩ), some of the lymphocyte signaling pathways controlled by these kinases can be redundant although they have distinct *in vivo* functions due to their broad substrate specificity. For example, in addition to NF-κB they are also involved in AP-1 or NFAT transcription factors activation in T cells (Tan and Parker, 2003). In this review we discuss the mechanisms by which aPKCs regulate T and B cell signaling after antigen stimulation and the role of the aPKCs-associated molecules Par-4, a potent inhibitor of aPKCs, and the scaffolding protein p62 in lymphocyte activation and differentiation.

### Atypical PKCs in B cell biology

B cells are lymphocytes originated from lymphoid precursors in the bone marrow after rearrangement of the immunoglobulin genes to provide the immune system with the specific repertoire of B cells to protect the body against pathogens (Harwood and Batista, 2010). B cells generation from hematopoietic precursors needs four different steps to take place: early pro-B cells, late pro-B cells, pre-B cells, and immature B cells formation. Before immunoglobulin gene rearrangement starts, the early pro-B cell subset emerges from the precursors giving rise to the following subset of late pro-B cells. In this step begins the rearrangement of D and J gene segments to generate pre-B cells with intact immunoglobulin heavy chains. When the rearrangement of the light-chain genes is completed the immature B cell subset is formed, expressing IgM on cell surface (B220^low^ MHC-II^high^ IgM^high^ IgD^low^). At this step, B cells undergo a selection process to eliminate self-reactive cells to avoid autoimmunity before going out to periphery. Once positioned in secondary lymphoid organs, immature B cells become transitional B cells that are ready to maturate within the follicles in lymph nodes or in the marginal zone of the spleen (marginal zone B cells). After antigen challenge, mature B cells (B220^high^ MHC-II^high^ IgM^low^ IgD^high^) become antibody-secreting plasma cells in germinal centers or memory B cells distributed elsewhere (Hardy and Hayakawa, 2001). The role of PKCs in these processes as well as in the activation and function of mature B cells is from diverse nature. Is important to remark that, unlike what is described for other PKCs, PKCδ is involved in the generation of B cell tolerance and anergy (Mecklenbrauker et al., 2002; Miyamoto et al., 2002) whereas PKCβ and PKCζ have a different role in B cell biology. Mice deficient for PKCβ has defective B cell signaling (Leitges et al., 1996) and PKCζ deficiency is associated to defects in B cell development, proliferation, and survival (Leitges et al., 2001; Martin et al., 2002; Moscat et al., 2003).

#### Role of PKCζ in secondary lymphoid organ maturation and B cell differentiation

Although the lack of PKCζ in mice generates no apparent abnormality, the formation, and maturation of secondary lymphoid organs is altered during the first weeks after birth. In this regard, 2–4 weeks old mice fail to develop an appropriate number of Peyer’s patches (PP) with an important reduction in follicles size and total cell number within each PP. Moreover, internal structure of PPs is also impaired as B and T cell zones are disrupted, this is due in part to the almost total lack of follicular dendritic cells (FDCs) that organized follicles structure. B cell zones are reduced and there is an imbalance between B220^high^ MHC-II^high^ IgM^low^ IgD^high^ mature and B220^low^ MHC-II^high^ IgM^high^ IgD^low^ immature B cells that is also observed in adult mice (Leitges et al., 2001). Regarding the spleen of these mice, although grossly normal, they present a defect in the formation of B cell follicles in the white pulp. The deficiency of FDCs in this organ induces disruption of the marginal zone architecture. Accordingly there is a decrease in the percentage of mature BCs in lymph nodes. However most of these defects are overcome in adult animals indicating that PKCζ could have an important role in B cell maturation in young animals that can be compensated by other molecules in adult mice (Leitges et al., 2001). In this regard it is important to note that B cell subpopulations in spleens and lymph nodes are normal and also microarchitecture of these organs has no apparent defects in adult animals.

#### B cell activation and survival is dependent of PKCζ signaling

**Figure 1:**
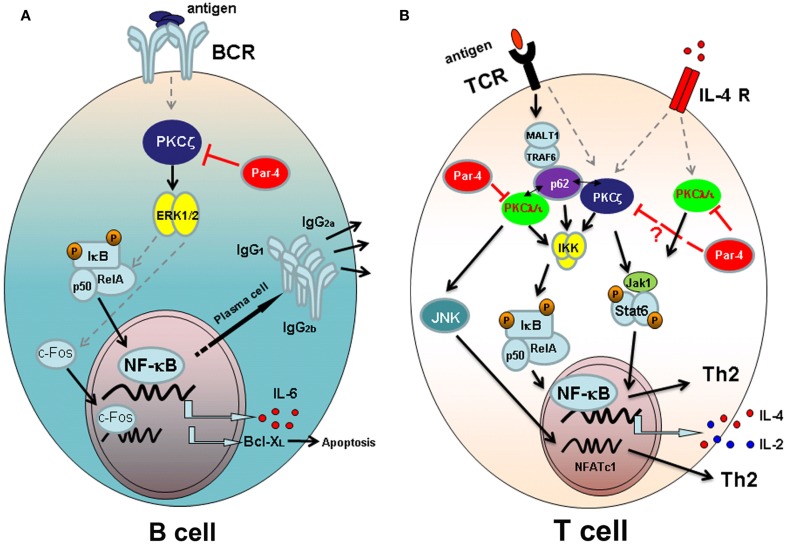
**Atypical PKC signaling pathways leading to lymphocyte activation and/or differentiation**. **(A)** Signaling through B cell antigen receptor (BCR) in B-lymphocytes. PKCζ is required for the activation of ERK after antigen challenge and necessary for IκB transcription and for the activation and nuclear translocation of c-Fos. The transcription of NF-κB-dependent genes IL-6 and Bcl-X_L_, and the secretion of T-dependent immunoglobulins depend on PKCζ-mediated signaling. The aPKC inhibitor Par-4 blocks PKCζ activation and B cell proliferation. **(B)** Signaling pathways in T lymphocytes. After antigen presentation, downstream signaling through T cell receptor (TCR) takes place. Atypical PKCζ and PKCλ/ɩ are important for IKK activation and NF-κB nuclear translocation, together with p62 scaffold protein that links the aPKCs with TCR activation through MALT-1 and TRAF-6 proteins. Moreover PKCλ/ɩ activates JNK and NFATc1 nuclear translocation, required for Th2 differentiation of T cells. Signaling through IL-4 receptor (IL-4R) requires both atypical PKCs to fully activate Jak1/Stat6 pathway and therefore to Th2 differentiation. Par 4 inhibits TCR and IL-4R signaling pathways blocking the activation of aPKCs.

In depth study of the role of PKCζ in B cell signaling indicates that this aPKC is required for an optimal survival rate, cell cycle entry, and proliferation after B cell receptor (BCR) stimulation in adult animals (Martin et al., 2002). As stated before, B cell subpopulation and lymphoid organ architecture of 4–6 weeks old animals is quite normal. In contrast to that observed in embryonic fibroblasts (EFs; Leitges et al., 2001), studies with PKCζ-deficient mice indicate that the defects in B cell activation and proliferation correspond to impaired activation of extracellular signal-regulated kinase (ERK), whereas the activation of other mitogen-activated protein kinases (MAPK) is not altered. Regarding transcription factors activation through PKCζ, a small decrease in activating protein-1 (AP-1) transcription factor activation is observed along with an inhibition of c-Fos induction in PKCζ-deficient mice (Martin et al., 2002; Figure 1A). Although PKCζ is an important mediator of the NF-κB pathway (Leitges et al., 2001), the activation and nuclear translocation of this transcription factor is not inhibited in BCs from PKCζ-deficient mice. However, activation of I*k*B transcription is severely impaired in PKCζ-deficient B cells after BCR stimulation and more importantly, transcription of NF-κB-dependent genes such as IL-6 or Bcl-X_L_ (important for B cell survival) is also inhibited (Figure 1A). These data demonstrate that although PKCζ is important for the transcription of NF-κB-dependent genes associated with proliferation and survival, and consistent with previous data of EF from PKCζ-deficient mice (Leitges et al., 2001), NF-κB nuclear translocation is not affected in the absence of this molecule in B cells (Martin et al., 2002).

#### Adaptive immune responses are altered in PKCζ-deficient mice

PKCζ is an essential kinase for B cell survival and function through the activation of I*k*B and Bcl-X_L_ transcription (Martin et al., 2002). These data agree with the notion that Rel, RelA, and NF-κB1 transcription factors are essential in B cell development, survival, proliferation, and immunoglobulin expression (Grossmann et al., 2000; Gugasyan et al., 2000). The lack of Rel/NF-κB genes or the individual subunits of the IκB kinase, IKKα and IKKβ, alters the development of adaptive immune response (Gerondakis et al., 1999). Accordingly, although PKCζ-deficient mice develop immune responses against T-independent and -dependent antigens and are able to induce the formation of germinal centers in the spleen, the T-dependent humoral immune responses are very faint. The secretion of IgG_1_, IgG_2a_, and IgG_2b_ immunoglobulins, specific for dinitrophenyl-ovalbumin (DNP-OVA) T-dependent antigen, is almost abrogated in PKCζ-deficient mice, indicating that these mice are not able to mount a proper humoral response against T-dependent antigens (Martin et al., 2002; Figure 1A).

Even nowadays there is little information on the role of the other family member of aPKCs, the PKCλ/ɩ, in secondary lymphoid organ development or signaling downstream BCR in mature B cells. This is in part due to the lack of viable homozygous deficient mice for PKCλ/ɩ as these mice die before birth (Soloff et al., 2004; Yang et al., 2009), which is incompatible with the study of mature B cells. While more effort needs to be done to clarify this issue, PKCλ/ɩ seems not to be activated after BCR stimulation or after incubation with anti-CD40 indicating that this aPKC could not be playing any role in B cell biology (Martin et al., 2002). In summary, PKCζ plays a major role in lymphoid organ formation and B cell differentiation and function that could be non-redundant with the other aPKCs, the PKCλ/ɩ.

#### Role of PKCλ in B cell development

The role of PKCλ/ɩ in BC development or function has not been studied in such detail so as PKCζ. However it is known that PKCλ is involved in pre-BCR-induced activation of NF-κB in B cells undergoing maturation process (Saijo et al., 2003). Src-family protein tyrosine kinases (SFKs) have an important role in the first steps during B cell development, controlling the generation, and survival of pre-B cells (Wechsler and Monroe, 1995). These effects are mediated by NF-κB thus in the absence of SFKs, IKK phosphorylation is inhibited and the nuclear translocation of NF-κB is impaired inducing defects in the formation of pre-B cells. It has been proposed that the atypical PKCλ is a mediator of SFK induced activation of NF-κB in immature B cells, since PKCλ activation in pro-B cells deficient for SFK restores IKK activation and NF-κB-mediated signaling (Saijo et al., 2003). The mechanisms of IKK activation by PKCλ are not addressed, however it could be a direct association by the consensus phosphorylation site Ser177 (Lallena et al., 1999), or by an indirect interaction with Bcl10 and Carma1 (Gaide et al., 2002).

### T cell signaling and atypical PKCs

The nature of effector T lymphocytes and their functional properties into inflammatory tissues has been a matter of study during decades. However, in the last few years the paradigms of effector cell populations at the site of inflammation has changed considerably with the emergence of new players. After the infection of invading pathogens or during inflammation, T cell activation, and subsequent differentiation of T helper lymphocyte subsets are crucial for the development of immune responses. Once the antigen presenting cell presents the antigen to naïve CD4 T cells, these cells experienced differentiation toward Th1, Th2, or Th17 T helper cell subsets in the lymph nodes. Afterward, T helper cells migrate to the inflamed tissue and develop an adaptive immune response. Th1 cells are characterized by the expression of T-bet and the release of the Th1-related cytokine IFN-γ. Th1 cells are involved in the generation of autoimmunity and inflammatory disorders mediated by cellular immune responses such as the clearance of pathogens. The effector subset Th2 is essential for the development of inflammatory conditions mediated by antibodies and in the generation of allergic responses such as asthma. Th2 cells produce mainly IL-4, IL-5, and IL-13 among other cytokines and express the transcription factors GATA-3, Stat6, and c-Maf. And the more recently discovered T helper subset, the Th17 cells are characterized by the synthesis of IL-17A, IL-17F, and IL-22. Th17 cell emerged as an independent differentiation pathway as the express a different panel of transcription factors such as Stat3 and RORγt (Harrington et al., 2005; Bettelli et al., 2007; Nakayamada et al., 2012). The role of classical and novel PKCs in T cell receptor (TCR) signaling and T cell differentiation into helper T cells have been addressed extensively. PKCþθ is recruited to the T cell synapse after TCR stimulation, what makes this PKC essential for the downstream signaling through NF-κB (Liu et al., 2000). The function of this PKC in T cell signaling is shared with PKCα, since both PKCs mediate T lymphocyte activation (Gruber et al., 2009). However PKCþθ have different roles in Th1, Th2, or Th17 differentiation. PKCþθ-deficient mice develop normal Th1 responses toward intracellular pathogens whereas Th2 asthmatic response against Helminth infection is impaired. Regarding Th17 responses, PKCþθ is required for the development of IL-17-driven experimental autoimmune encephalomyelitis (Marsland and Kopf, 2008).

#### PKCζ and T helper differentiation

In contrast to other PKCs, such as PKCþθ, essential for T cell activation (Sun et al., 2000), PKCζ-deficient T cells show normal proliferation and survival rates after TCR stimulation with anti-CD3 and anti-CD28 antibodies (Martin et al., 2002). Further studies on the differentiation of T helper subsets have revealed that Th1 differentiation from PKCζ-deficient naïve CD4^+^ T cells appear not to be affected in this function, as the release of the typical Th1-associated cytokine IFN-γ is not affected by the lack of PKCζ. However PKCζ is definitely necessary for the differentiation of naïve T cells into Th2 cells (Martin et al., 2005; Figure 1B). PKCζ-deficient CD4^+^ T cells differentiated *in vitro* under Th2 polarizing conditions secrete low levels of Th2-related cytokines IL-4, IL-5, IL-10, and IL-13, compared to the amount of cytokines released by CD4^+^ T cells from WT mice. These defects in Th2 differentiation are due to the almost completely lack of nuclear translocation and activation of GATA-3, Stat6, RelA, c-Maf, and NFATc1, all of them essential transcription factor for Th2 polarization (Zhu et al., 2010). The main cytokine produced by Th2 cells is IL-4 and exerts its function through the IL-4 receptor and the subsequent activation of the downstream Jak1/Stat6 signaling pathway. Importantly, PKCζ interacts with and phosphorylates Jak1 hence controlling the IL-4/Stat6 signaling pathway in T cells (Duran et al., 2004a; Figure 1B). As a consequence PKCζ-deficient mice develop a mild form of concanavalin A-induced hepatitis, a disease dependent of T cell activation through the IL-4/Stat6 pathway (Jaruga et al., 2003), with a severe decrease in leukocyte infiltration and liver apoptosis (Duran et al., 2004a). Moreover PKCζ is an important player in the Jak1/Stat6 signaling cascade involved in the activation through IL-4 and in Th2 differentiation. In this regard Th2 cells play an important role in humoral immunity against extracellular pathogens and in allergic reactions. Consequently, Th2 responses are inhibited in OVA-induced allergic airway inflammation in PKCζ-deficient mice. The adoptive transfer of PKCζ-expressing Th2 cells from WT mice to PKCζ-deficient mice induces severe inflammation in the lungs indicating that PKCζ is a critical molecule in the mechanisms of Th2 induction of inflammation in the airways after antigen exposure (Martin et al., 2005).

#### PKCλ/ɩ and T helper differentiation

The atypicals PKCζ and PKCλ/ɩ share a significant structural homology (Diaz-Meco and Moscat, 2012) however, both PKCs play a different role in the activation and proliferation of naïve T cells. In contrast to PKCζ-deficient T lymphocytes, PKCλ/ɩ-deficient naïve T cells, isolated from conditional PKCλ/ɩ knockout (KO) mouse in which PKCλ/ɩ is specifically deleted in activated T cells by crossing PKCλ/ɩ^fl/fl^ with Cre*^OX40^* mice, have impaired proliferation after TCR stimulation (Martin et al., 2005; Yang et al., 2009). Moreover, PKCλ/ɩ-deficient CD4^+^ T cells secrete low levels of Th2 typical cytokines IL-4 and IL-13 whereas the secretion of IFN-γ by these cells is completely normal after TCR stimulation with anti-CD3 and anti-CD28 antibodies (Yang et al., 2009). Interestingly, the addition of IL-4 to PKCλ/ɩ-deficient T cells cultured under Th2 differentiation conditions restores the ability to secrete IL-4 and IL-13 cytokines by polarized PKCλ/ɩ-deficient Th2 cells. Therefore, while PKCλ/ɩ is important for the proliferation and Th2-cytokine secretion by naïve T cells, PKCζ is critical for Th2 development and function (Martin et al., 2002; Yang et al., 2009; Figure 1B).

However, other important aspects for the development of immune responses *in vivo* are shared by these two aPKCs. The levels of expression of both proteins are very similar in lung tissue (Leitges et al., 2001; Martin et al., 2002) and are much more expressed in Th2 cells than in Th1 or non-skewing Th0 cells (Martin et al., 2002; Yang et al., 2009). Accordingly to PKCζ-deficient mice, PKCλ/ɩ^fl/fl^ Cre*^OX40^* mice have decreased allergic airway inflammation in lungs after OVA challenge, although this inhibition of asthmatic response is more dramatic under PKCλ/ɩ deficiency (Yang et al., 2009). Conditional PKCλ/ɩ-deficient mouse is unable to recruit eosinophils to the lungs and also have very low levels of IL-4 and IL-13 Th2-related cytokines in the broncho-alveolar lavage (BAL) fluid, compared to WT mice. This is consistent with significantly reduced levels of IgE specific for OVA in the serum of PKCλ/ɩ^fl/fl^ Cre*^OX40^* mice challenged with OVA. This is another feature shared with PKCζ-deficient mouse, defects in airway inflammation in this model of allergic asthma are due to an deregulation in the activation and nuclear translocation of GATA-3, NFATc1, p65, and Stat6 in T cells (Yang et al., 2009).

### Partners of aPKCs in T cell activation and adaptive immune responses

The two isoforms of aPKCs, ζ and λ (ɩ in humans) are highly homologous at the amino acid level (Selbie et al., 1993; Akimoto et al., 1994). aPKC amino-terminal domain encodes several protein interaction sequences including a PB1 domain (Phox and Bem1p), a protein–protein interaction domain shared by other adapter proteins such as p62, Par-6, or NBR1 (Moscat and Diaz-Meco, 2000; Ito et al., 2001), and the C1 domain that interacts with the protein Par-4, an inhibitor of aPKCs enzymatic activity, by a zinc-finger domain (Diaz-Meco et al., 1996). Together with the aPKCs, p62 and Par-4 interacting proteins have an important role in the control of T cell activation and in the development of adaptive immune responses.

#### Par-4, an inhibitor of aPKCs, controls the strength of B and T cell responses

The aPKC inhibitor Par-4 plays an important role in the modulation of apoptosis through the regulation of NF-κB activation (Garcia-Cao et al., 2003). Par-4 inhibitor was first described in prostate cancer cells and linked to neoplastic alterations in the prostate (Sells et al., 1994; Barradas et al., 1999) moreover it is known that this protein plays also an important role in B and T cell activation and proliferation. Par-4 deficient mice develop spontaneous splenomegaly, although present normal proportions of T and B cells in primary and secondary lymphoid organs in steady state (Lafuente et al., 2003). However, B and T cells isolated from Par-4 deficient mice have an increased proliferation rate after antigen receptor stimulation compared to WT mice, indicating that this protein acts at the level of antigen receptor signaling. Interestingly Par-4 deficient T cells secrete high amounts of IL-2 *in vitro* after stimulation. As Par-4 is a pro-apoptotic protein, Par-4 deficient T cells are more resistant to apoptosis induced cell death than their WT T cell control (Lafuente et al., 2003). As it was previously mentioned, aPKCs downstream signaling cascade controls NF-κB activation (Leitges et al., 2001) and, in turn, Par-4 inhibits this pathway and proliferation of T cells through the inhibition of aPKCs phosphorylation. Then, it seems that Par-4-mediated inhibition of T cell proliferation is not controlled through PKCζ since the proliferation of T cells from PKCζ-deficient mice is normal (Martin et al., 2002). Hence Par-4 should exert its effect through the other aPKC, the PKCλ/ɩ. Moreover, primary EFs from Par-4-deficient mice have decreased JNK activation that is observed also in T cells from these mice (Garcia-Cao et al., 2003; Lafuente et al., 2003). JNK is a potent inhibitor of NFATc1 transcription factor essential for T cell differentiation and function and accordingly Par-4 deficient T cells have an increase in NFATc1 activation, which may explain the high levels of IL-2 secretion by these cells that could directly influence the high rate of proliferation of Par-4-deficient T cells (Chow et al., 2000; Crabtree and Olson, 2002). A well as PKCζ and PKCλ/ɩ play an important role in T cell differentiation toward helper subsets, Par-4 is important for the control of Th2 responses. Par-4 deficiency leads to aPKC hyperactivation and subsequent inhibition of JNK, signaling pathway required for the control of IL-4 secretion, and Th2 differentiation (Dong et al., 1998; Figure 1B). Moreover, *in vivo* experiments in a model of T cell-dependent hepatitis have demonstrated that Par-4 deficiency leads to an increase in IL-4 signaling pathway, increasing the levels of Th2-cytokines, eotaxin, apoptosis, and liver injury after treatment with Con-A. These results are consistent with an enhanced signaling downstream IL-4 in EFs from Par-4 deficient mice, where Jak1 and Stat6 phosphorylation is increased demonstrating a connection with an enhanced signaling through aPKC (Duran et al., 2004a; Martin et al., 2005; Yang et al., 2009).

#### aPKC scaffold protein p62 controls T helper differentiation

The adapter protein p62 interacts with aPKCs through their PB1 protein interaction domain and is essential in the maintenance of NF-κB activity during osteoclastogenesis (Duran et al., 2004b). Similarly is a key protein for the long-term NF-κB activation after TCR stimulation of naïve CD4^+^ T cells (Martin et al., 2006). Unlike PKCζ and PKCλ/ɩ do, p62 controls Th2 differentiation and function by an IL-4 independent mechanism. Th2 cells deficient for p62 have impaired activation and nuclear translocation of GATA-3 and RelA, playing an important role in NF-κB activation, whereas Stat6 signaling through IL-4 receptor is not affected (Das et al., 2001; Martin et al., 2006). In this regard, proximal TCR downstream signaling pathway is not altered in the absence of p62 as Zap-70, ERK, or AKT activation and Ca^2+^ flux are normal after antigen receptor triggering. Degradation of IκBα, an early indicator of NF-κB activation, is also intact in p62-deficient cells while IκBα activation is inhibited several hours after TCR triggering along with NF-κB inhibition. Interestingly, p62 expression is induced at later time points after TCR stimulation and particularly under Th2 differentiation conditions. This is possible because p62 establish a connection with Malt-1 and TRAF6, both proteins involved in IKK ubiquitination (Wooten et al., 2005), favoring NF-κB activation after long-term TCR stimulation probably through PKCλ/ɩ (Figure 1B). Accordingly with impaired Th2 differentiation *in vitro*, p62-deficient mice also show a dramatically reduction of lung inflammation toward OVA-induced allergic airway disease demonstrating a key role of p62 in Th2 function *in vivo* (Martin et al., 2006).

## Concluding Remarks

It is clear that over the recent years the aPKCs have emerged as critical players in the control of T and B cell function, which has important implications as potential therapeutic targets in inflammation and immunity. Importantly due to their role also in cancer, they can also be considered critical players in the ability of the tumor cell to grow and proliferate but also as key players in the tumor microenvironment whereby they can be central in orchestration signals controlling the impact that inflammation has in tumor progression and initiation. Future studies using the animal models described here will be instrumental in addressing these important questions.

## Conflict of Interest Statement

The authors declare that the research was conducted in the absence of any commercial or financial relationships that could be construed as a potential conflict of interest.
